# Influence of Tap Water Quality and Household Water Use Activities on Indoor Air and Internal Dose Levels of Trihalomethanes

**DOI:** 10.1289/ehp.7141

**Published:** 2005-03-24

**Authors:** John R. Nuckols, David L. Ashley, Christopher Lyu, Sydney M. Gordon, Alison F. Hinckley, Philip Singer

**Affiliations:** ^1^Department of Environmental and Radiological Health Sciences, Colorado State University, Fort Collins, Colorado, USA; ^2^Emergency Response and Air Toxicants Branch, Centers for Disease Control and Prevention, Atlanta, Georgia, USA; ^3^Battelle, Centers for Public Health Research and Evaluation, Durham, North Carolina, USA; ^4^Battelle, Columbus, Ohio, USA; ^5^Department of Environmental Sciences and Engineering, University of North Carolina, Chapel Hill, North Carolina, USA

**Keywords:** biomarkers, chlorination, disinfection by-products, exposure, trihalomethane, water use

## Abstract

Individual exposure to trihalomethanes (THMs) in tap water can occur through ingestion, inhalation, or dermal exposure. Studies indicate that activities associated with inhaled or dermal exposure routes result in a greater increase in blood THM concentration than does ingestion. We measured blood and exhaled air concentrations of THM as biomarkers of exposure to participants conducting 14 common household water use activities, including ingestion of hot and cold tap water beverages, showering, clothes washing, hand washing, bathing, dish washing, and indirect shower exposure. We conducted our study at a single residence in each of two water utility service areas, one with relatively high and the other low total THM in the residence tap water. To maintain a consistent exposure environment for seven participants, we controlled water use activities, exposure time, air exchange, water flow and temperature, and nonstudy THM sources to the indoor air. We collected reference samples for water supply and air (pre–water use activity), as well as tap water and ambient air samples. We collected blood samples before and after each activity and exhaled breath samples at baseline and postactivity. All hot water use activities yielded a 2-fold increase in blood or breath THM concentrations for at least one individual. The greatest observed increase in blood and exhaled breath THM concentration in any participant was due to showering (direct and indirect), bathing, and hand dishwashing. Average increase in blood THM concentration ranged from 57 to 358 pg/mL due to these activities. More research is needed to determine whether acute and frequent exposures to THM at these concentrations have public health implications. Further research is also needed in designing epidemiologic studies that minimize data collection burden yet maximize accuracy in classification of dermal and inhalation THM exposure during hot water use activities.

Trihalomethanes (THMs) are a by-product of water chlorination, arising from the reaction between natural organic matter in the source water and chlorine used for disinfection. There are four primary species of THM: chloroform (CHCl_3_), bromodichloromethane (CHBrCl_2_), dibromochloromethane (CHBr_2_Cl), and bromoform (CHBr_3_). The speciation of the THM depends on raw water quality and treatment characteristics (Miles et al. 2002). The U.S. Environmental Protection Agency (EPA) has established a maximum contaminant level of 0.08 mg/L for the total THM (TTHM) because of increased evidence of adverse health effects linked to these compounds (U.S. EPA 1998). Researchers have found an association between elevated levels of THM and adverse health outcomes, including cancer (Cantor et al. 1978, 1987, 1998; Hildesheim et al. 1997; King and Marrett 1996; McGeehin et al. 1993) and adverse reproductive outcomes (Aschengrau et al. 1989, 1993; Bove et al. 1995; Gallagher et al. 1998; Klotz and Pyrch 1999; Waller et al. 1998). Exposure assessments for most of these studies were based on reported levels of TTHM in the water distribution system serving the participants’ residences, and in some cases reconstructing study participants’ water consumption histories.

Exposure to THM through routes other than ingestion has been demonstrated as a significant component of the overall exposure matrix. In controlled experiments, Weisel et al. (1992) and Xu and Weisel (2005) reported elevated breath concentrations of CHCl_3_ due to showering. In a later field study of 33 subjects using public water supplies in New Jersey with relatively low THM concentrations, Weisel et al. (1999) determined that timing of sampling postshower exhaled air was important in order to capture a high correlation to water concentration. Critical time frames reported by their study were 20 min for CHCl_3_ and CHBrCl_2_ and 5 min for CHBr_2_Cl and CHBr_3_.

Weisel and Jo (1996) demonstrated that dermal contact is an important route of exposure for CHCl_3_, reporting higher exhaled air concentrations from this route than from inhalation due to showering and bathing. Gordon et al. (1998) also reported elevated CHCl_3_ concentrations in exhaled breath from subjects who breathed clean air while bathing in waters ranging in temperature from 30 to 40°C (86–104°F). For these dermal-only exposures, they reported that for similar levels of CHCl_3_ in the bath water, much higher levels of the compound in exhaled air were measured from an individual taking a 40°C bath compared with the same individual taking a 30 or 35°C bath.

Studies have demonstrated that exposure to THM results in significant increases in blood THM concentrations. Backer et al. (2000) reported increases in blood CHCl_3_, CHBrCl_2_, and CHBr_2_Cl compared with pre-activity blood levels in groups of approximately 10 individuals each due to showering, bathing, and consuming 1 L of cold tap water for a 10-min period. They found that increases in blood concentrations of these THMs from showering or bathing were significantly greater than the increases from drinking 1 L of water. Pegram et al. (2002) reported maximum blood concentrations of CHBrCl_2_ ranging from 0.4 to 4 ng/mL due to ingestion versus 39–170 ng/mL due to dermal contact with water containing the same concentration of CHBrCl_2_. They also reported that blood CHBrCl_2_ levels returned much more rapidly to baseline after ingestion (4 hr) as opposed to after dermal exposure (24 hr). Lynberg et al. (2001) measured THM in pre- and postshower blood samples from 25 participants in each of two water utility service areas. They reported significant intersite differences in both tap water samples and blood THM levels, as well as significant increases in blood THM levels for all participants due to the showering event. Miles et al. (2002) further analyzed the data from the field study and found that although showering activity shifted the THM distribution in the blood toward that found in the corresponding tap water (including concentration), there was no significant correlation between blood concentration and tap water concentration.

Household water uses other than showering and bathing have not been evaluated in terms of potential exposure to THM. In this study, we determined the relative contributions of showering and bathing, along with 12 other water use activities, to THM exposure in a household environment. The purpose of this article is to provide a description of the methods used in our study and a summary of the results. The findings are relevant to the design and implementation of epidemiologic studies concerning exposure to volatile water supply contaminants.

## Materials and Methods

### Study location/participants.

We conducted our study at a single residence in each of two sites: one in North Carolina (NC site) and the other in Texas (TX site). The floor plans for the study residences at the NC site and TX site were almost identical. Both were three-bedroom/two-bathroom, one-story, ranch-style houses (about 111.5 m^2^ or 1,200 ft^2^ total floor space). Both residences had central heating, ventilation, and air conditioning (HVAC) systems, and both had electric water heaters. Each residence was served by a public water distribution system. The study was conducted 5 August through 17 September 2002 in North Carolina and 13 October through 6 November 2002 in Texas. We treated the data as representative of a water supply with relatively high (NC) and relatively low (TX) THM concentrations, predominated by chlorinated THM species.

We planned for and recruited seven participants by advertising in local media and distributing study flyers on local college campuses. We used a standardized questionnaire to screen applicants for the following eligibility criteria (acceptable range given in parentheses): age (18–35 years), body mass index (22–24), tobacco smoking (nonsmoker only), alcohol consumption (average < 2 drinks/day), and swimming activity (< 4 days/week). We also excluded applicants who reported asthma or other breathing problems, high blood pressure or hypertension, a history of problems associated with blood draws, regularly taking any medications for any health conditions, or any condition that would prevent them from conducting the water use activities prescribed by our study. The final study group was composed of three males and one female at the NC site, and one male and two females at the TX site. The age range for participants in our study was 21–30 years. Two of the male participants at the NC site reported their race as African American. All other participants reported their race as Caucasian.

### Data collection.

Before the introduction of participants, we prepared the study residence for data collection and analysis. Only one of the bathrooms in each residence was used as the study bathroom. Approximately 30 min before the first activity began each day, the second bathroom door was shut and the vent fan turned on. To prevent and account for contribution of THMs to household air, the use of the second bathroom during the study activities was minimized as much as possible and was documented. The showerhead in the study bathroom of each residence was replaced with a custom showerhead designed to maintain consistent flow. This showerhead was connected to a remote water sampling apparatus designed to minimize loss of volatile THM. The apparatus was used to collect water samples from the showerhead and the shower stall drain. The thermostat for the HVAC in each house was set at 75°F, and the HVAC fan was set to the “on” position during the entire study period. The exhaust fan in the study bathroom was not turned on at any time during the study. At each study site, we conducted airflow and tracer gas studies to characterize the house-to-environment air exchange rates and bathroom-to-house air flow rates and to identify the optimal locations for collecting household air samples during the THM exposure study (Dietz and Cote 1982).

We collected data THM exposure data over a 2-day period for each study participant. The second day of the study typically occurred approximately 1 week after the first. On each day, the participant performed a set of prescribed water use activities while we collected pre- and postactivity samples of air, water, blood, and exhaled breath. These activities are listed in Table 1. Between events on the participation day, the participant was required to remain in the residence. We designed the sampling regimen so that activities expected to result in the largest increase in internal dose levels were spaced at estimated time intervals sufficient to allow blood THM concentrations to return as much as possible to preexposure levels before the next water use activity. For some activities we collected concurrent air and/or water samples, as well as exhaled breath samples. Water temperature was measured during each activity.

To reduce the likelihood of inadvertent THM exposure, each participant arrived at the study residence the night before his/her scheduled day of data collection and slept in the study residence. Upon arrival, the participant completed a questionnaire to provide information on demographics, water use and consumption in the past 48 hr, and exposure to chemicals that might be confounding factors in the study. These data were collected primarily to screen for water or chlorinated compound use (e.g., swimming) that could interfere with our premise that early morning blood concentrations could represent a “baseline” for each individual. The subjects were instructed to wear swimsuits for the showering and bathing components of the study.

Over the study period, we measured the flow of water to each study house using a water meter data logger (Meter Master model 100EL; F.S. Brainard Company, Burlington, NJ). These data were collected primarily for modeling purposes and will be discussed in a separate report. We measured ambient and indoor temperatures and relative humidity using electronic thermometers. We controlled and standardized the water temperature for each study activity.

### Water samples.

We collected 21 water samples over the 2-day period. These samples were either associated with a water use activity or collected from a cold-water tap over the course of each exposure day to establish “baseline” THM concentrations (TTHM and each of four species). We collected and analyzed duplicates of each sample. All water samples were collected using headspace-free 40-mL acid-washed glass vials. Immediately after collection, ammonium sulfate was added to the sample in order to quench residual chlorine and prevent further THM formation. We measured and recorded the temperature of the tap water for each sample. Sample containers were refrigerated and packed into coolers with ice packs and shipped by overnight express courier to the University of North Carolina at Chapel Hill for analysis using gas chromatography.

### Air samples.

We collected air samples to determine the levels of THM (TTHM and each of the four species) in the air associated with each activity. Thirteen samples were collected over the 2-day study period for each participant. We collected a “baseline” sample each day before any water use activity. The air samples were collected using precleaned and evacuated SUMMA polished 6-L stainless steel canisters (Scientific Instrumentation Specialists, Moscow, ID, and Biospherics, Hillsboro, OR). We collected “grab” samples by opening the canister valve and allowing air to flow into the canister until atmospheric pressure equilibrium was attained (≤ 1 min). We shipped exposed canisters by overnight express courier to Battelle Memorial Institute (Columbus, OH) for analysis. Samples were analyzed by automated gas chromatography/mass spectrometry (GC/MS) using a modified version of U.S. EPA Method TO-14 (Winberry et al. 1990).

### Blood samples.

We collected blood samples from each participant in order to examine the levels of THM (TTHM and each of four species) associated with each water use activity. Vacutainers (Becton, Dickinson & Co., Franklin Lakes, NJ) were prepared by heating, restoration of vacuum, and resterilization in order to eliminate background contamination from the blood collection device (Cardinali et al. 1995). We collected samples approximately 5 min before and after each activity, using a multisample adapter (venous catheter). Additional blood samples were collected 30 min after the shower and bath activities. The catheter remained in the participant for the duration of each day of the study, approximately 12 hr. We collected a total of 26 10-mL blood samples from each participant over the course of the 2-day study, 14 on day 1 and 12 on day 2. After collection, each blood sample was refrigerated and packed into coolers with ice packs, and at the end of each day shipped by overnight express courier to the Volatile Organics Laboratory at the Centers for Disease Control and Prevention (CDC; Atlanta, GA). We analyzed THM in the blood samples using a variation of the standardized method reported by Ashley et al. (1992). This method includes spiking 3-mL blood samples with isotopically labeled standards, extracting with solid-phase micro-extraction, and analysis by GC followed by high-resolution magnetic-sector MS. We quantified blood THM concentrations using calibration curves generated from dilutions of pure samples of each THM species. Blanks and quality control materials were analyzed with each analytical run. Detection limits were in the parts per quadrillion range, allowing the quantification of most samples even at background levels.

### Breath samples.

We collected breath samples using a self-administered procedure in which the subject exhaled alveolar air directly into an evacuated single breath canister (Pleil and Lindstrom 1995). For this study we used 1-L Silcosteel stainless steel canisters (Entech, Simi Valley, CA) fitted with a short Teflon tube that served as a disposable mouthpiece. We instructed the subject to begin sample collection near the end of a normal resting tidal breath in order to provide what is mostly alveolar breath. We collected a total of 15 breath samples from each subject over the 2-day study period. Baseline measurements were obtained once per day before all activities began. Samples were shipped at the end of each day by overnight express courier to Battelle for THM analysis (TTHM and each of four species), which was carried out by the same automated GC/MS procedure used for air samples.

### Data analysis.

We calculated summary statistics (mean, SD, median, range) for measured THM species in water, air, blood, and exhaled breath samples and for measurements of temperature in the water samples and in the ambient air during activities. We calculated relative exposure, defined as the ratio between pre- and postactivity blood concentration and between exhaled breath concentrations, for each participant and activity. We plotted the data and examined for natural break points. Based on this procedure, we established a cut-point of 2-fold deviation from baseline concentrations as indicators of meaningful increase or decrease in these biologic marker concentrations. We established similar criteria of ± 20% for the ratio of activity-related water concentrations to baseline (cold tap) water sample concentrations, and a 5-fold deviation in the ratio of activity-related air concentrations to baseline. Our approach is similar to that suggested by the American Chemical Society Committee on Environmental Improvement (ACSCEI) to determine whether increases in biologic concentrations are meaningful when comparing environmental chemistry data (ACSCEI 1980). The ACSCEI suggested an increase of at least three times the SD of the smallest (baseline) concentration in making this determination. Our approach is generally more conservative.

We used a repeated measures design of the general linear model (Ott and Longnecker 2001) to test for statistically significant inter-site, interparticipant, and temporal differences in measured water temperature and concentrations of THM in water. We used two-factor experiments with repeated measures on one factor (order of activity or baseline measurements as a proxy for time), and α = 0.05 level of significance, to conduct these analyses.

## Results

### Water supply temperature and THM concentration.

**Figure 1:**
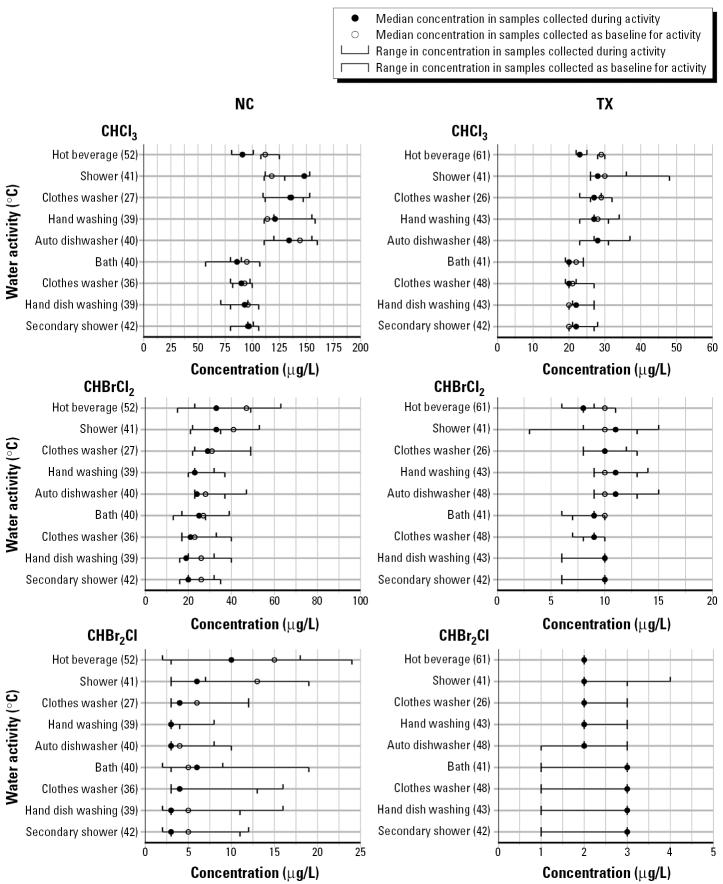
on a graph, that in the samples collected as baseline for the activity. All concentrations are rounded to nearest integer for presentation purposes. The concentration scales used vary by study site and THM compound.

Figure 1 provides a summary of the median and range of concentrations of THM measured in baseline (cold tap) and water samples from each water use activity that resulted in at least a 2-fold increase in biologic markers of exposure for at least one participant. It also includes the median water temperature for each sample type. The only activity that did not meet the criteria for inclusion in Figure 1 was ingestion of a cold tap water beverage.

Baseline THM concentrations in the tap water were much higher at the NC site for TTHM, ranging from 113 to 212 μg/L compared with a range of 12–53 μg/L at the TX site. Although these concentrations did change over the course of the day, the difference between concentration for any THM by time of day was not statistically significant across the study population (*p* = 0.07–0.65). THM concentrations in the activity-associated water were also much higher at the NC site compared with the TX site.

Most ratios of THM concentration in activity-associated water to concentration in baseline (cold tap water) samples were near or below 1.0. Only the ratio for CHCl_3_ for the showering event at the NC site exceeded our criteria of a 20% increase as being meaningful. At the NC site, median ratios of activity to baseline concentration for several THM species and activities were at least 20% less than 1.0, including CHBrCl_2_ in showering and bathing, and CHBr_2_Cl in showering and hand dish-washing. At the TX site, we did not observe a deviation of > 20% in ratios of THM concentration in activity and baseline water samples at the group or individual level, except for one participant whose water for the shower and for hand dish washing had a ratio of 3.2 for CHBr_2_Cl and 3.3 for TTHM, respectively.

We found that activity-associated water temperatures for most activities in Figure 1 were much higher than the temperature of the corresponding baseline water sample, with the exception of the automatic clothes washing activity. Median temperatures of the baseline (cold tap) water samples were very similar, with a difference of < 2°C for any activity between the two study sites. The intersite differences in the median water temperature were < 1°C for most activities. We found no statistically significant correlation between water temperature and THM concentration, with the exception of CHBrCl_2_ at the NC site (*p* = 0.02).

### Air temperature and THM concentration.

Table 2 provides a summary of median and range of concentrations of THM measured in baseline samples (before any water use activities) and in ambient air samples for each water use activity that resulted in at least a 2-fold increase in biologic markers of exposure for at least one participant. It also includes the median and range in air temperature for each sample type. The only activity that did not meet the criteria for inclusion in Table 2 was ingestion of a cold tap water beverage.

At both study sites, we observed a > 5-fold increase in the ratio of activity ambient air to baseline THM concentration for all THM compounds other than CHBr_3_ for participants as a group due to showering and indirect shower exposure, and due to the bathing activity (except CHBr_2_Cl). The air TTHM concentration during showering increased by 70% across individuals at the NC site and by 38% at the TX site (data not shown). We observed a 4- to 11-fold (median = 7) increase in ambient air TTHM concentration due to the hand washing activity across participants at the NC site. This increase was primarily due to a corresponding increase in CHCl_3_ concentration. We also observed large increases in ambient air CHCl_3_ due to the automatic clothes washing with bleach (median increase > 9-fold) and the hand dish washing (median > 5-fold) activities across participants at the TX site. For most of the other water use activities listed in Table 2, we observed a slight to moderate increase in ambient air THM concentration at both sites (median increase < 2.5-fold).

For the activities listed in Table 2, median temperatures of the baseline ambient air samples were equal for day 1 and within 0.7°C for day 2. Median temperatures of ambient air during the water use activities were within 5% of baseline at both sites, except for the clothes washing II activity at the TX site. For that activity, the median air temperature was 27°C (81°F) compared with a median baseline temperature of 23°C (73°F).

### Markers of exposure: blood and exhaled air THM.

Table 3 provides a summary of median and range of concentrations of THM measured in blood samples collected 5 min before and after each water-related activity by study site. At both sites, there was a > 2-fold increase in blood concentrations for all participants and all THM species except CHBr_3_ due to the showering and bathing activities. Increases as a result of showering were 5- to 15-fold in participants at the NC site and approximately 5-fold at the TX site. Increases as a result of the bathing activity were 3- to 6-fold in participants at the NC site and 3- to 19-fold at the TX site. Hand dish washing resulted in a 2- to 8-fold increase in blood THM concentrations (except CHBr_3_) in two of the three participants at the Texas site. Increases of 3-fold in concentrations of CHBrCl_2_ and CHBr_2_Cl were observed in the other participant. Hand dish washing resulted in a < 2-fold increase in blood THM concentrations in three of the four participants at the NC site.

The average preshower blood TTHM concentrations at the NC and TX sites were 47 and 19 pg/mL, respectively. The average increases in blood TTHM due to showering at the sites were 358 and 79 pg/mL, respectively. We observed similar preactivity average blood TTHM concentrations for bathing and hand dish washing (except one participant at the TX site). The average increases in concentration for bathing were 164 and 118 pg/mL at the NC and TX sites, respectively. The average increases in concentration for hand dish washing were 98 and 57 pg/mL, respectively, but there was a high degree of interparticipant variation at both sites. Increases in blood THM for the other activities were generally < 20 pg/mL and highly varied.

Table 4 provides a summary of the median and range of concentrations of THM in exhaled breath samples collected before all water use activities (baseline) and during or after activities by study site. The baseline exhaled breath THM concentrations were very similar between the two sites for all THM species except CHCl_3_, which was consistently higher at the NC site. Baseline CHBrCl_2_ concentration in one NC participant was 9 μg/m^3^, but this was inconsistent with all other baseline measurements at the NC site, which ranged from below the detection limit (0.8) to 4.6 μg/m^3^.

We found a > 2-fold increase in the median exhaled breath concentrations of TTHM across participants as a group due to bathing (both study sites) and showering (NC) activities, and an almost 2-fold increase due to showering at the TX site. These increases in TTHM were primarily due to increases in CHCl_3_ concentration. Similar increases in median exhaled breath concentrations of CHCl_3_ were also observed due to hand dish washing activities at both sites, the automatic dish washing activity at the TX site, and the automatic clothes washing with bleach activity at the NC site. Across individual participants, increases in exhaled breath TTHM concentrations due to showering ranged from 3- to 6-fold at the NC site and were approximately 2-fold at the TX site. Individual increases due to bathing ranged from 3- to 6-fold at the NC site and 3- to 19-fold at the Texas site. Individual increases due to hand dish washing ranged from approximately 1.5- to 2.5-fold at both sites, except for one outlier at the NC site with a measured decrease of 0.5-fold. This outlier had no influence on any of the reported results. We observed a 2-fold or better increase in the exhaled breath concentration of at least one THM compound in at least one study participant due to each of the other water use activities, with the exception of hand washing and indirect shower exposure.

## Discussion

We measured blood and exhaled air concentrations of THM as biomarkers of exposure to participants conducting 14 common household water use activities (Table 1). We found that the showering (10 min) and bathing (20 min) activities consistently resulted in at least 2-fold increases in median blood and exhaled breath TTHM across two study groups, regardless of whether the study site was characterized by high (NC site median = 136 μg/L) or low (TX site median = 38 μg/L) TTHM in the residential water supply. This magnitude of increase was observed for all THM species except CHBr_3_ in the blood samples, but only for CHCl_3_ in the exhaled breath samples. We also observed > 2-fold increases in median exhaled breath concentrations of CHCl_3_ at both sites and in blood CHCl_3_ and TTHM in two of the three participants at the TX site for the hand dish washing activities. There was no activity without a 2-fold increase in concentration in any biomarker of exposure for at least one THM and one individual.

The greatest observed increase in blood and exhaled breath THM concentration in any participant was due to showering and bathing. The average increases in blood TTHM due to showering were 358 and 79 pg/mL at the NC and TX sites, respectively. Average increases due to bathing were 164 and 118 pg/mL, and those due to hand dish washing were 98 and 57 pg/mL, respectively. However, we observed a high degree of interparticipant variation in the increase due to hand dish washing at both sites. Increases in blood TTHM concentration due to other activities were < 20 pg/mL and were also highly variable. More human-based research is needed to determine whether acute and frequent exposures to THM at these concentrations have public health implications.

The results of our study are consistent with findings of other studies for which shower water and pre- and postshower blood THM concentrations have been reported. Table 5 presents a summary of shower water and participant blood (pre- and postshower) THM concentrations for two studies in addition to ours. If we group the shower water concentrations of CHBrCl_2_ for the five study sites described in Table 5 into three categories: 6, 11–14, and 33 μg/L, the corresponding median blood CHBrCl_2_ concentrations reported for these groups are 19, 28–43, and 93 pg/mL after showering for 10 min. These findings indicate a dose response between concentration in the source water and blood. Similar correspondence between shower water and postshower blood CHBr_2_Cl and CHCl_3_ concentrations were observed across the five study sites, as well as for source water and postbathing THM concentrations reported for our study and by Backer et al. (2000). Lynberg et al. (2001) did not conduct a bathing analysis.

Our observations are also consistent with results reported in other residential studies of exposures to disinfected tap water in which air and exhaled breath samples were analyzed for THM. Table 6 summarizes results of during-shower air THM concentrations from three studies (Egorov et al. 2003; Kerger et al. 2000; May et al. 1995) in addition to ours. THM concentrations of exhaled breath from participants during showering were also reported by Egorov et al. (2003). In all cases reported in Table 6, the air concentrations during showers showed the same decreasing trend of CHCl_3_ > CHBrCl_2_ > CHBr_2_Cl, which was consistent with their relative concentrations in the source water of each respective study.

When we adjust for variation in THM water concentrations across the studies by taking the ratios of the shower air to source water concentrations, this ratio is approximately 2.2 and 2.4 μg/m^3^ per microgram per liter water for the “high” and “low” sites in our study, respectively. In comparison, we obtained a ratio of 1.7 from the May et al. (1995) and Egorov et al. (2003) data and a ratio of 3.5 from the Kerger et al. (2000) data. The differences in ratios between these studies could be due to a variety of factors known to affect THM transfer coefficients from water to air that we did not take into account in this comparison. These factors include water temperature and flow rate, shower duration, volume of shower enclosure, air exchange rates, and showerhead type. Available published studies on the measurement of THM concentrations in exhaled breath are sparse. Table 6 summarizes our results for the “high” and “low” sites along with values presented by Egorov et al. (2003) from their study of exposures to tap water disinfection by-products in a Russian city. In each case, the data for the three THMs listed show a corresponding gradient, high to low, between the during-shower air concentrations and the postshower exhaled breath concentrations. However, both our “high” site and “low” site concentrations for breath CHCl_3_ are significantly lower than the value reported by Egorov et al. (2003) despite the relatively close agreement between air concentrations at our “high” site and their value (Table 6). A reason for the observed differences could be the time when the samples were taken after exposure ended (Gordon et al. 1998; Weisel et al. 1999; Xu and Weisel 2005). In our study, breath samples were taken 5 min after exposure ceased; Egorov et al. (2003) collected breath samples within 1 min after subjects completed their showering activity.

We observed changes in baseline (cold tap water) THM concentrations over the course of each study day. However, the difference between baseline concentration for any THM by time of day was not statistically significant across the study population (*p* = 0.07–0.65). We also observed a high degree of variation between tap water THM concentrations over the period of study, especially at the NC site. For example, at this site water samples were collected 7 different days over the period of approximately 43 days; the range in TTHM concentrations in the samples collected at 0800 hr on each of those days was 139–200 μg/L (average = 169 μg/L), and the maximum CHBrCl_2_ was 63 μg/L (range, 23–63 μg/L). The THM levels in our samples were much different from the average concentrations reported by the utility that provides water to our NC study site. For example, the utility reported an annual average TTHM concentration of 76.7 μg/L (range, 28–145 μg/L) and a maximum CHBrCl_2_ concentration of 17 μg/L (range, 5–17 μg/L) for the year in which our study was conducted. These findings are important in terms of exposure assessment for epidemiologic studies concerning THM, because they indicate that although “snapshot” measurements of THM on a given day can be representative of levels for water use activities on that day, they may not be representative of THM in a specific residential water supply over a longer period of time.

The results of the present study support the findings of other studies that blood THM concentrations in response to equal or equivalent THM exposure appear to be higher in some individuals. At each of our study sites, we observed a large difference in relative increase in THM blood levels by one of the study participants in response to exposure by showering in waters with approximately the same THM concentration and temperature. We also observed differences in response for the same individual to exposure from hand dishwashing. Although our sample size is very small, these findings lend support to similar patterns reported by Backer et al. (2000) and Lynberg et al. (2001). Backer et al. (2000) suggested that such differentiation in response may be the result of differences in individuals’ abilities to metabolize THM. A number of metabolic enzymes exist in polymorphic form. For example, some THM are substrates for glutathione *S*-transferase theta-1 (GSTT1)–mediated glutathione conjugation reactions (Landi et al. 1999). Among Caucasian populations, about 17–18% of people are null for this gene. Another candidate enzyme is CYP2E1, which has a demonstrated role in metabolism of THM (Allis et al. 2001; Constan et al. 1999). Further research is needed to understand the implication of these findings in terms of design of epidemiology studies concerning THMs.

Our findings in the present study have important ramifications for exposure assessment in epidemiologic studies concerning THMs. The study confirms that showering and bathing activities are important sources of THM exposure. It provides evidence that hand dishwashing, indirect shower exposure, and other hot water use activities could also be important sources but need more study. Water temperature, THM concentration, and duration of use have been demonstrated to be important variables for quantifying THM exposure during showering and bathing (Giardino and Andelman 1996; Keating et al. 1997; Kerger et al. 2000; Wilkes et al. 2004). Water temperature was not correlated to water THM concentration in the present study. It is well established that THM concentrations of water in residential water heaters are generally much higher than in tap water from the utility distribution system, and we observed much higher temperatures in activity-associated water compared with baseline (cold tap) samples. However, we observed THM concentration ratios (TTHM and all species) near or below 1.0 between these water samples for most all activities. THM concentrations in air samples collected in association with these water use activities were all significantly elevated, indicating that THMs formed by heating of the water supply were volatile. For example, showering and indirect shower exposure median air concentrations were 318 and 142 μg/m^3^ compared with a baseline of 4 and 3 μg/m^3^, respectively at our NC site (Table 2). The fact that the ratios of the shower air to source water concentrations for the “high” and “low” sites were about equal (2.2 and 2.4) in our study indicates that estimates of air THM concentrations associated with specific hot water use activities may be possible if accurate THM water concentrations are known.

Weisel and Chen (1994) observed a doubling of CHCl_3_ concentration and a 50% increase in CHBrCl_2_ and CHBr_2_Cl in water heated to 65°C that contained 0.7–0.8 mg/L total chlorine residual. They reported that most of this increase occurred within 0.5 hr and was essentially complete within 1 hr. If THM concentrations do “plateau” in a residential water heater, obtaining measurements of temperature and THM concentration in separate hot and cold water samples during an epidemiology study could simplify exposure assessment. The temperature measurements could be used to estimate potential range of dermal exposure. Gordon et al. (1998) reported a strong effect of bath water temperature on dermal absorption of CHCl_3_, and it is likely this effect would hold for other hot water uses with dermal contact. Likewise, it might be possible to estimate air THM concentrations for specific water use activities based on the hot and cold water THM concentration. These results could be used in conjunction with air to water THM concentration ratios to construct “confidence intervals” for predictions of air THM concentrations from specific water use activities. A limitation to this approach is that these ratios can vary by activity as a function of room volume, ventilation, and other factors. For example, in our study intersite differences in these factors were minimized for the shower activity, and the ratios were near equal (2.2 and 2.4). However, the average air to water CHCl_3_ concentration ratios for the bathing activities, which were measured in the bathroom rather than shower stall, were 0.7 at our NC site and 1.2 at the TX site. The intersite difference in ratios for the bathing activity was due to a difference in bathroom volume. More research is needed to determine if standardized air to water THM concentration ratios for hot water activities related to significant THM exposure can be developed and applied in the context of an epidemiologic study.

The results of the present study clearly indicate that epidemiology studies concerning THMs need to consider hot water use activities as important exposure events. Further research is needed in designing epidemiologic studies that minimize data collection burden yet maximize accuracy in classification of dermal and inhalation THM exposure during these activities.

## Figures and Tables

**Table 1:** Description of water use activities and duration over the course of the study.

Time	Water use activity	Duration (min)
Day 1		
2100*^a^*	Participant arrives at the study house and sleeps there overnight	
0800	Baseline measurements: ambient household air, tap water, blood THM	6.0
0820	Breakfast, including preparation and consumption of a hot beverage from tap water (0.25 L)	25.0
1000	Hot water shower*^b^*	13.0*^c^*
1300	Lunch, including drinking 0.5 L of cold tap water	30.0
1500	Automatic clothes washing (clothes washer)*^d^*	50.0
1730	Hand washing*^e^*	0.5
1800	Supper, including consumption of bottled water (no specified volume)*^f^*	45.0
1900	Automatic dish washing, open dishwasher at end of cycle	50.0
2100	Participant departs study house	
Day 2 (1 week after day 1)		
2100*^a^*	Participant arrives at the study house and sleeps there overnight	
0800	Baseline measurements: ambient household air, tap water, blood THM	6.0
0820	Breakfast, including consumption of a cold beverage prepared from tap water (0.25 L)	25.0
1000	Hot water bath*^b^*	23.0*^g^*
1300	Lunch, including consumption of bottled water (no specified volume)*^f^*	30.0
1400	Automatic clothes washing, adding bleach during the wash cycle	50.0
	(clothes washer II)*^d^*	
1600	Hand washing of dishes*^h^*	10.0
1800	Supper, including consumption of bottled water (no specified volume)*^f^*	45.0
1900	Sitting in room adjacent to the study bathroom and a shower event, opening bathroom door at end of the event*^i^*	13.0
2100	Participant departs study house	

a Evening before day of study; arrival between 2100 and 2300 hr allowed.

b No cleaning products such as soap or shampoo were used by the participant; subjects wore swimsuits.

c Participant in shower stall or bath for 10 min, followed by 3 min in study bathroom with door closed for changing clothes.

d Participant did not stay in same room as water use device.

e No cleaning products such as soap were used by the participant.

f Bottled water was tested and confirmed to have no THM species present.

g Filling time from 1000 to 1006 hr, maintained constant (6 min) for each participant; this was sufficient volume to submerge the torso and legs; participant stayed in the tub from 1006 to 1020 hr (14 min), followed by 3 min in study bathroom with door closed for changing clothes; subjects wore swimsuits.

h Detergent (Dawn Ultra; Procter & Gamble, Cincinnati, OH) was used.

i Termed “indirect shower exposure.”

**Table 2:** Median temperature and concentration of THM in air (μg/m^3^) for baseline and activities with at least a 2-fold increase in blood concentration for at least one participant.

	Air temp (°C)		CHCl_3_		CHBrCl_2_		CHBr_2_Cl		CHBr_3_		TTHM	
Activity	NC site	TX site	NC site	TX site	NC site	TX site	NC site	TX site	NC site	TX site	NC site	TX site
Baseline day 1	24 (22–24)	24 (23–25)	4 (2–10)	2 (1–2)	3 (BDL–7)	2 (2–3)	BDL (—)	BDL (—)	BDL (—)	BDL (—)	8 (5–19)	5 (5–7)
Hot beverage	24 (24–25)	23 (23–24)	7 (3–10)	2 (2–2)	2 (1–4)	3 (2–3)	BDL (—)	BDL (—)	BDL (—)	BDL (—)	10 (6–16)	6 (6–7)
Shower	25 (24–32)	24 (20–28)	318 (219–351)	67 (50–70)	54 (31–68)	23 (20–25)	9 (4–13)	4 (3–6)	BDL (—)	BDL (—)	384 (255–431)	95 (74–102)
Clothes washer	24 (24–27)	27 (25–27)	21 (7–25)	4 (2–5)	7 (BDL–8)	2 (0.7–3)	BDL (BDL–2)	BDL (—)	BDL (—)	BDL (—)	31 (9–34)	4 (2–5)
Hand washing	24 (22–27)	23 (22–23)	49 (19–85)	3 (3–5)	10 (3–13)	2 (1.3–2.3)	2 (BDL–2)	BDL (—)	BDL (—)	BDL (—)	62 (23–101)	6 (6–9)
Automatic dishwasher	24 (24–25)	25 (24–26)	8 (4–12)	5 (4–5)	2 (BDL–3)	3 (3–3)	BDL (—)	BDL (—)	BDL (—)	BDL (—)	11 (6–18)	9 (9–10)
Baseline day 2	24 (23–24)	23 (21–24)	3 (2–4)	1 (0.8–2)	1 (BDL–1)	1 (1–3)	BDL (—)	BDL (—)	BDL (—)	BDL (—)	6 (4–7)	4 (4–7)
Bath	24 (22–24)	23 (21–24)	71 (49–98)	14 (8–61)	12 (9–14)	7 (4–15)	2 (1–3)	1.4 (BDL–2)	BDL (—)	BDL (—)	88 (60–112)	24 (13–79)
Clothes washer II	24 (24–25)	27 (27–28)	9 (8–33)	9 (4–13)	2 (1–5)	2 (0.9–3)	BDL (—)	BDL (—)	BDL (—)	BDL (—)	12 (11–39)	14 (6–17)
Hand dish washing	24 (24–25)	24 (24–28)	8 (6–17)	5 (3–9)	2 (1–4)	1 (1–5)	BDL (—)	BDL (—)	BDL (—)	BDL (—)	11 (9–23)	8 (6–15)
Indirect shower exposure	24 (22–25)	24 (22–24)	142 (117–370)	75 (63–86)	30 (20–114)	27 (25–29)	7 (3–11)	5 (3–7)	BDL (—)	BDL (—)	176 (151–495)	108 (100–115)

BDL, below detection limit (detection limits are 0.5 μg/m^3^ for CHCl_3_, 0.7 μg/m^3^ for CHBrCl_2_, 0.8 μg/m^3^ for CHBr_2_Cl, and 1.0 μg/m^3^ for CHBr_3_). Values shown in parentheses are ranges; ranges are not included if all samples were at or below detection.

**Table 3:** Median THM concentration in blood (pg/mL) approximately 5 min before and after water use activities.

	CHCl_3_				CHBrCl_2_				CHBr_2_Cl				CHBr_3_				TTHM			
	NC site		TX site		NC site		TX site		NC site		TX site		NC site		TX site		NC site		TX site	
Activity	Pre	Post	Pre	Post	Pre	Post	Pre	Post	Pre	Post	Pre	Post	Pre	Post	Pre	Post	Pre	Post	Pre	Post
Hot bev	40 (34–44)	31 (30–36)	19 (8–22)	13 (9–16)	9 (6–17)	8 (5–15)	4 (4–8)	3 (3–9)	2 (1–5)	2 (0.8–5)	2 (1–4)	1 (1–4)	0.6 (0.5–1)	0.6 (0.5–1)	0.5 (0.5–0.7)	0.6 (0.5–0.8)	52 (41–64)	44 (36–52)	28 (14–32)	21 (13–26)
Shower	26 (23–83)	290 (262–374)	13 (11–13)	63 (56–66)	6 (3–8)	93 (64–95)	4 (3–7)	28 (26–31)	1 (0.6–3)	13 (12–18)	1 (0.9–3)	6 (6–10)	0.7 (0.5–1)	0.8 (0.5–1)	0.5 (0.5–0.6)	0.7 (0.6–1)	34 (31–90)	399 (338–482)	18 (16–23)	97 (88–108)
Lunch w/water	51 (38–99)	45 (43–54)	37 (18–44)	41 (33–41)	11 (9–14)	12 (9–13)	6 (5–12)	7 (5–9)	2 (2–3)	3 (2–3)	2 (1–5)	2 (1–4)	0.6 (0.5–1)	0.6 (0.5–1)	0.6 (0.5–0.8)	0.6 (0.6–0.7)	66 (51–110)	59 (57–70)	45 (25–62)	48 (47–51)
Clothes washer I	32 (30–44)	52 (51–166)	27 (19–43)	35 (19–45)	7 (5–9)	12 (8–14)	5 (4–9)	5 (2–8)	2 (1–2)	2 (1–3)	2 (1–4)	2 (0.8–4)	0.6 (0.5–0.9)	0.5 (0.5–0.8)	0.6 (0.5–0.6)	0.5 (0.5–0.7)	43 (39–50)	67 (66–175)	35 (25–56)	42 (22–58)
Hand washing	36 (27–48)	48 (34–51)	23 (17–33)	19 (11–43)	9 (5–10)	11 (6–13)	4 (3–8)	5 (3–8)	2 (0.8–2)	2 (0.9–3)	1 (0.9–3)	1 (0.8–3)	0.5 (0.5–0.6)	0.6 (0.5–0.6)	0.6 (0.5–0.7)	0.6 (0.5–0.6)	47 (33–61)	61 (41–65)	29 (21–39)	25 (15–31)
Auto dishwasher	32 (22–36)	38 (30–43)	17 (14–43)	29 (17–39)	8 (4–9)	9 (6–11)	4 (3–4)	4 (4–4)	2 (0.7–2)	2 (0.8–3)	1 (0.9–5)	1 (1–3)	0.6 (0.6–0.6)	0.5 (0.5–0.6)	0.6 (0.5–1)	0.5 (0.5–0.5)	42 (27–47)	49 (37–56)	21 (20–62)	40 (22–45)
Cold bev	30 (24–95)	40 (29–56)	21 (20–50)	24 (16–85)	7 (3–47)	6 (5–24)	5 (4–8)	4 (3–9)	2 (0.5–17)	2 (0.8–9)	1 (1.0–3)	1 (0.6–3)	0.6 (0.5–0.9)	0.5 (0.5–0.8)	0.5 (0.5–0.6)	0.5 (0.5–0.5)	39 (29–161)	48 (37–88)	27 (26–62)	36 (21–89)
Bath	37 (27–40)	161 (125–188)	12 (8–22)	54 (48–156)	5 (5–14)	41 (40–43)	3 (2–7)	36 (26–65)	1 (1–5)	10 (6–13)	1 (0.5–3)	10 (8–11)	0.6 (0.5–0.9)	0.7 (0.5–1)	0.5 (0.5–0.5)	1 (0.5–1)	44 (35–60)	212 (181–234)	16 (12–32)	101 (83–231)
Clothes washer II	33 (22–44)	52 (38–61)	22 (12–39)	17 (—)*a*	5 (5–12)	8 (8–14)	8 (4–8)	5 (5–8)	2 (0.8–3)	2 (1–4)	2 (0.9–3)	2 (1–2)	0.5 (0.5–0.8)	0.6 (0.5–1)	0.5 (0.5–0.5)	0.5 (0.5–0.6)	44 (30–50)	66 (50–72)	34 (18–50)	17 (7–24)
Hand dish washing	43 (39–48)	73 (41–285)	33 (9–41)	42 (25–97)	7 (5–15)	19 (8–63)	4 (3–9)	12 (7–66)	2 (0.7–4)	6 (2–11)	1 (0.5–3)	3 (1.1–18.1)	0.6 (0.5–1)	0.6 (0.5–0.7)	0.6 (0.5–0.6)	0.7 (0.5–2)	56 (45–60)	99 (52–359)	38 (13–53)	58 (33–183)
Indirect shower exposure	35 (28–43)	50 (45–59)	52 (15–52)	19 (12–61)	6 (5–11)	10 (6–15)	5 (3–9)	6 (3–9)	1 (1–4)	2 (0.8–4)	1 (0.6–3)	2 (0.6–3)	0.6 (0.5–0.6)	0.5 (0.5–1)	0.5 (0.5–0.5)	0.5 (0.5–0.6)	45 (36–53)	63 (53–70)	53 (21–57)	23 (19–73)

Abbreviations: Auto, automatic; bev, beverage; w/, with. Values shown in parentheses are ranges.

a One participant with blood concentration of 17 pg/mL.

**Table 4:** Median and range of THM concentrations (μg/m^3^) in exhaled air: baseline and post-water activity by study site.

	CHCl_3_		CHBrCl_2_		CHBr_2_Cl		CHBr_3_		TTHM	
Activity	NC site	TX site	NC site	TX site	NC site	TX site	NC site	TX site	NC site	TX site
Baseline day 1	5 (2–6)	1 (1–2)	2 (BDL–5)	2 (2–3)	BDL (—)	BDL (—)	BDL (—)	BDL (—)	9 (4–13)	6 (5–6)
Hot beverage	4 (2–5)	2 (0.8–5)	2 (BDL–5)	3 (1–4)	BDL (—)	BDL (—)	BDL (—)	BDL (—)	7 (5–14)	7 (6–8)
Shower	24 (16–51)	6 (5–8)	6 (2–8)	3 (3–4)	BDL (—)	BDL (—)	BDL (—)	BDL (—)	28 (26–61)	11 (9–14)
Clothes washer	11 (3–17)	1 (0.7–2)	3 (BDL–6)	1 (BDL–2)	BDL (—)	BDL (—)	BDL (—)	BDL (—)	15 (6–25)	4 (4–5)
Hand washing	6 (3–11)	1 (0.9–1)	2 (BDL–2)	2 (1–5)	BDL (—)	BDL (—)	BDL (—)	BDL (—)	9 (5–15)	5 (4–12)
Automatic dishwasher	4 (2–4)	3 (3–4)	1 (BDL–2)	2 (2–2)	BDL (—)	BDL (—)	BDL (—)	BDL (—)	7 (5–15)	5 (4–12)
Baseline day 2	5 (2–12)	1 (BDL–2)	2 (1–9)	0.7 (BDL–2)	BDL (—)	BDL (—)	BDL (—)	BDL (—)	9 (6–15)	4 (3–6)
Bath	15 (11–22)	7 (4–9)	3 (1–4)	3 (3–3)	BDL (—)	BDL (—)	BDL (—)	BDL (—)	20 (14–26)	13 (9–13)
Clothes washer II	12 (6–13)	2 (2–3.5)	2 (1–8)	2 (1–2)	BDL (—)	BDL (—)	BDL (—)	BDL (—)	16 (9–46)	6 (5–7)
Hand dish washing	14 (5–18)	3 (3–4)	2 (BDL–3)	2 (1–5)	BDL (—)	BDL (—)	BDL (—)	BDL (—)	18 (7–22)	7 (6–11)
Indirect shower exposure	5 (2–8)	2 (1–2)	0.8 (BDL–2)	2 (2–2)	BDL (—)	BDL (—)	BDL (—)	BDL (—)	8 (4–11)	6 (5–6)

BDL, below detection limit (detection limits are 0.5 μg/m^3^ for CHCl_3_, 0.7 μg/m^3^ for CHBrCl_2_, 0.8 μg/m^3^ for CHBr_2_Cl, and 1.0 μg/m^3^ for CHBr_3_). Values shown in parentheses are ranges. Ranges are not included if all samples were at or below detection.

**Table 5:** Comparison of median shower water and pre- and postshower blood THM concentrations for participants in three studies.

	Shower water concentration (μg/L)					Postshower blood concentration (pg/mL)*^a^*					Ratio of post- to preshower blood concentration				
	Backer et al. 2000	Lynberg et al. 2001		Present study		Backer et al. 2000	Lynberg et al. 2001		Present study		Backer et al. 2000	Lynberg et al. 2001		Present study	
THM compound*^b^*		High site	Low site	High site	Low site		High site	Low site	High site	Low site		High site	Low site	High site	Low site
CHCl_3_	28	85	8	148	28	120	280	57	290	63	4	3	7	2	2
CHBrCl_2_	6	14	12	33	11	21	38	43	93	28	4	3	4	3	3
CHBr_2_Cl	1	14	2	6	2	5	41	6	13	6	5	3	3	2	3

*n* = 11 in Backer et al. (2000); *n* = 25 at each site of Lynberg et al. (2001); and *n* = 4 and 3 at the high (NC) and low (TX) sites, respectively, in the present study.

a Approximately 10 min postshower.

b CHBr_3_ was above, below, or near detection limit in water source at four of five sites and thus was not comparable.

**Table 6:** Comparison of THM concentrations in source water, during-shower air, and postshower breath concentrations in this and other published studies.

	Source water concentration (μg/L)					During-shower air concentration (μg/m^3^)*^a^*					Postshower breath concentration (μg/m^3^)*^b^*				
THM compound	May et al. 1995*^c^*	Kerger et al. 2000	Egorov et al. 2003	Present study		May et al. 1995*^c^*	Kerger et al. 2000	Egorov et al. 2003	Present study		May et al. 1995*^c^*	Kerger et al. 2000	Egorov et al. 2003	Present study	
				High site	Low site				High site	Low site				High site	Low site
CHCl_3_	51	47	198	148	28	84	165	330	318	67	—	—	110	24	6
CHBrCl_2_	17	42	7	33	11	24	80	8	54	23	—	—	1	6	3
CHBr_2_Cl	6	31	1	6	2	ND	16	ND	9	4	—	—	ND	1	1

ND, not determined. Kerger et al. (2000) and Egorov et al. (2003) reported mean concentrations; May et al. (1995) reported median concentrations; we report median concentrations from Tables 2, 4, and 5. *n* = 44 in May et al. study; *n* = 20 for source water and *n* = 12 for shower air in Kerger et al. study; *n* = 14 for source water, *n* = 35 for shower air, and *n* = 9 for exhaled breath in Egorov et al. study; *n* = 4 and 3 for source water, shower air, and exhaled breath at the high (NC) and low (TX) sites, respectively, in the present study. In water source, CHBr_3_ was near or below limit of detection at most sites; in air samples, CHBr_2_Cl and CHBr_3_ were below limits of detection in Egorov et al. and May et al. studies; in breath samples, CHBr_2_Cl and CHBr_3_ were below limits of detection in Egorov et al. and the present study.

a Shower duration: May et al. reported 10 min; Kerger et al. reported 6.8 min and 12 min; Egorov et al. reported 15–20 min; we report 10 min.

b Breath sample collection time: Egorov et al. reported ≤ 1 min postexposure; we report 5 min postexposure.

c Median values for CHCl_3_, CHBrCl_2_, and CHBr_2_Cl for source water and shower air estimated from plots in May et al.
